# A real-world disproportionality analysis of Everolimus: data mining of the public version of FDA adverse event reporting system

**DOI:** 10.3389/fphar.2024.1333662

**Published:** 2024-03-12

**Authors:** Bin Zhao, Yumei Fu, Shichao Cui, Xiangning Chen, Shu Liu, Lan Luo

**Affiliations:** ^1^ Xiamen Health and Medical Big Data Center, Xiamen, China; ^2^ Xiamen Medicine Research Institute, Xiamen, China; ^3^ Department of Breast Surgery, The Affiliated Hospital of Guizhou Medical University, Guiyang, China; ^4^ NHC Key Laboratory of Male Reproduction and Genetics, Guangdong Provincial Reproductive Science Institute (Guangdong Provincial Fertility Hospital), Guangzhou, China; ^5^ Department of Medical Quality Control, Sun Yat-sen University Cancer Center, Guangzhou, China

**Keywords:** Everolimus, data mining, FAERS, pharmacovigilance, adverse event

## Abstract

**Background:** Everolimus is an inhibitor of the mammalian target of rapamycin and is used to treat various tumors. The presented study aimed to evaluate the Everolimus-associated adverse events (AEs) through data mining of the US Food and Drug Administration Adverse Event Reporting System (FAERS).

**Methods:** The AE records were selected by searching the FDA Adverse Event Reporting System database from the first quarter of 2009 to the first quarter of 2022. Potential adverse event signals were mined using the disproportionality analysis, including reporting odds ratio the proportional reporting ratio the Bayesian confidence propagation neural network and the empirical Bayes geometric mean and MedDRA was used to systematically classify the results.

**Results:** A total of 24,575 AE reports of Everolimus were obtained using data from the FAERS database, and Everolimus-induced AEs occurrence targeted 24 system organ classes after conforming to the four algorithms simultaneously. The common significant SOCs were identified, included benign, malignant and unspecified neoplasms, reproductive system and breast disorders, *etc.* The significant AEs were then mapped to preferred terms such as stomatitis, pneumonitis and impaired insulin secretion, which have emerged in the study usually reported in patients with Everolimus. Of note, unexpected significant AEs, including biliary ischaemia, angiofibroma, and tuberous sclerosis complex were uncovered in the label.

**Conclusion:** This study provided novel insights into the monitoring, surveillance, and management of adverse drug reaction associated with Everolimus. The outcome of serious adverse events and the corresponding detection signals, as well as the unexpected significant adverse events signals are worthy of attention in order to improving clinical medication safety during treatment of Everolimus.

## 1 Introduction

The mammalian target of rapamycin (mTOR) is a serine/threonine kinase comprised of two functionally different protein complexes, mTOR complex 1 (mTORC1) and mTOR complex 2 (mTORC2) (Murugan, 2019). Evidence has demonstrated that mTOR regulates multiple targets and pathways simultaneously and is involved in various biological processes, including cell survival, growth, metabolism, protein synthesis, and autophagy (Tian et al., 2019). Current knowledge indicates that the dysregulation of mTOR signal is associated closely with cancer occurrence, making mTOR an attractive therapeutic target of cancer (Popova and Jücker, 2021). In recent years, a variety of mTOR inhibitors have been developed and proved to have great pharmacological activity (Zou et al., 2020). Everolimus is an orally available mTOR inhibitor, which can bind with the intracellular receptor FK506-binding protein (FKBP-12) and form the everolimus-FKBP-12 complex to block the activation of mTORC1 (Hua et al., 2019). Meanwhile, it also inhibits the expression of hypoxia-inducible factor 1 (HIF-1) and vascular endothelial growth factor (VEGF) (Kirchner et al., 2004), leading to the significant immunosuppressive and antiproliferative properties. Everolimus is already approved for: 1) hormone receptor (HR)- positive, human epidermal growth factor receptor 2 (HER2)-negative advanced breast cancer with exemestane in postmenopausal women (Du et al., 2018; Moreau-Bachelard et al., 2023), 2) advanced renal cell carcinoma after treatment with sorafenib or sunitinib therapy (Yangyun et al., 2022), 3) advanced pancreatic neuroendocrine tumors (panNET) (Chan et al., 2017), 4) renal angiomyolipoma or subependymal giant-cell astrocytoma (SEGA) in tuberous sclerosis complex (TSC) (Song et al., 2019), and has also been proposed for improving graft function in transplant patients (Tang et al., 2015). While generally well-tolerated with common adverse drug reactions (ADRs) such as stomatitis, anemia, hyperglycemia, and hyperlipidemia (Paplomata et al., 2013), some uncommon but potentially fatal adverse effects warrant specific attention, including non-infectious pneumonitis (Dabydeen et al., 2012) and gastrointestinal hemorrhage (Tsunematsu et al., 2019). Therapeutic drug monitoring adverse events (AEs) should be valued during the use of Everolimus, and more relevant real-world data is necessary for establishing clinically reasonable drug reference standards.

The Food and Drug Administration Adverse Event Reporting System (FAERS) is a publicly accessible database specifically designed to facilitate the FDA’s post-marketing safety monitoring of drugs and therapeutic biologic products (Yu et al., 2021). Reports were quantitatively assessed by signal detections, where a signal meant a drug-related AE. Researchers has begun to be analyzed on a variety of drugs and diseases using the FAERS database to find potential AEs that should be of particular concern, aiming to provide guidance on clinical medication and treatment (Yang et al., 2023; Zhao et al., 2023).

In the present study, we analyzed the Everolimus-related AEs reported from the first quarter of 2009 to the first quarter of 2022 through data mining of FAERS. In addition to discovering new AE signals, exploratory investigations on the current situation and update of its safety profile were also conducted. We believe that the research will contribute to rational and safe clinical therapeutic application of Everolimus.

## 2 Methods

### 2.1 Data collection

The FAERS is a publicly available database designed to support the post-marketing surveillance program for drugs and therapeutic biologics, including all adverse event information and medication error information collected directly from consumers and healthcare professionals. This real-world data associated with Everolimus’s AEs between the first quarter of 2009 and the last quarter of 2022 was extracted and preprocessed using MYSQL. Then the clean and standardized data were selected and mapped to MedDRA23.0 to remove the duplicated case records and identify the AEs. The significant AEs were mapped to preferred terms (PTs) and system organ class (SOC) based on the structural hierarchy of the MedDRA terminology (Mascolo et al., 2021). Data pertaining to clinical characteristics, including gender, age, and reported countries, were collected and compiled for analysis. Severe outcomes included life-threatening, hospitalization, disability, and death.

### 2.2 Statistical analysis

As a commonly used approach in pharmacovigilance study, disproportionality analysis was performed to detect spontaneous signals (Zink et al., 2013). Frequentist and Bayesian methods were employed to assess potential associations between everolimus and AEs using the reporting odds ratio (ROR), the proportional reporting ratio (PRR), the Bayesian confidence propagation neural network (BCPNN), and the empirical Bayes geometric mean (EBGM) (Song et al., 2020). Signals of AEs with at least 3 records were recorded for analysis, and the signals would be selected when conforming to the four algorithm criteria simultaneously, with lower limit of 95% confidence interval (CI) > 1.0. The statistical shrinkage method was employed to minimize the risk of spurious associations caused by rare events with low number of cases (Norén et al., 2013). All data processing and statistical analyses were performed using R software (version 4.0.2).

## 3 Result

### 3.1 General characteristics

From the first quarter of 2009 and the first quarter of 2022, a total of 24,575 reports on Everolimus were reported. The clinical characteristics of events with Everolimus are described in.

Table 1 Among all AEs, more female than male patients (54.85% vs. 36.35%) were reported. The largest percentage of reports (13.07%) were in patients aged 60–69 years. Elderly individuals (aged >60 years) accounted for a high proportion with 23.82% (n = 5,854). The country that reported the most was the United States (47.56%), followed by Japan (4.47%), Germany (4.12%), France (2.94%) and Canada (2.22%). Death (25.02%) was the most frequently reported severe outcome, followed by hospitalization and life-threatening occurring in 5,706 (23.22%) and 527 (2.14%) cases, respectively. The incidence of disability was the lowest (0.98%).

**TABLE 1:** The characteristics of reports associated with everolimus from the FAERS database (2009 Q1 to 2022 Q4).

	Everolimus	
	Counts	Percentages (%)
Number of events	24,575	
**Gender**		
Male	8,934	36.35
Female	13,479	54.85
Unknown	2,162	8.80
**Age**		
<20	697	2.84
20–29	330	1.34
30–39	485	1.97
40–49	996	4.05
50–59	2,337	9.51
60–69	3,213	13.07
70–79	2051	8.35
>80	590	2.40
Unknown	13,876	56.46
**Reported Countries (the top ranked)**		
US (United States)	11,687	47.56
JP (Japan)	1,099	4.47
DE (Germany)	1,013	4.12
FR (France)	723	2.94
CA (Canada)	546	2.22
**Serious Outcomes**		
Hospitalization	5,706	23.22
Death	6,148	25.02
Life-threatening	527	2.14
Disability	241	0.98

### 3.2 Signal detection

Signal strengths of reports of Everolimus at the System Organ Class (SOC) level are described in Table 2. Statistically, Everolimus-associated AEs occurrence targeted 24 organ systems. The significant SOCs were “Neoplasms benign, malignant and unspecified (incl cysts and polyps) (SOC: 10029104, 3515)”, “Reproductive system and breast disorders (SOC: 10038604, 211)”, “Injury, poisoning and procedural complications (SOC: 10022117, 213)”, “Musculoskeletal and connective tissue disorders (SOC: 10028395, 337)”, and “Congenital, familial and genetic disorders (SOC: 10010331, 5)”.

**TABLE 2:** Signal strength of AEs of Everolimus at the System Organ Class (SOC) level in FDA Adverse Event Reporting System (FAERS) source.

**SOC Code**	**SOC**	**Case Reports**	**ROR (95% CI)**	**PRR (95% CI)**	**χ2**	**IC (IC025)**	**EBGM (EBGM05)**
10022891	Investigations	3834	4.21 (4.06-4.36)	3.71 (3.6-3.82)	7843.61	1.88 (1.77)	3.68 (3.56)
10017947	Gastrointestinal disorders	4726	6.73 (6.52-6.95)	5.63 (5.48-5.77)	18361.74	2.48 (2.37)	5.56 (5.39)
10029104	Neoplasms benign, malignant and unspecified (incl cysts and polyps)	3515	11.85 (11.43-12.28)	10.29 (9.98-10.62)	29174.70	3.33 (3.21)	10.06 (9.71)
10038738	Respiratory, thoracic and mediastinal disorders	3588	4.85 (4.68-5.03)	4.29 (4.16-4.42)	9272.06	2.09 (1.97)	4.25 (4.11)
10038359	Renal and urinary disorders	892	5.28 (4.94-5.65)	5.12 (4.8-5.47)	2944.70	2.34 (2.12)	5.07 (4.74)
10018065	General disorders and administration site conditions	7492	4.92 (4.79-5.06)	3.72 (3.65-3.8)	16125.32	1.89 (1.8)	3.7 (3.6)
10042613	Surgical and medical procedures	306	3.49 (3.11-3.9)	3.46 (3.09-3.86)	531.45	1.78 (1.4)	3.44 (3.07)
10019805	Hepatobiliary disorders	575	8.68 (7.99-9.44)	8.5 (7.84-9.22)	3737.76	3.06 (2.78)	8.35 (7.68)
10021881	Infections and infestations	201	4.23 (3.68-4.86)	4.2 (3.66-4.82)	485.94	2.06 (1.59)	4.17 (3.62)
10038604	Reproductive system and breast disorders	211	10.27 (8.95-11.78)	10.19 (8.89-11.67)	1706.62	3.32 (2.86)	9.96 (8.68)
10005329	Blood and lymphatic system disorders	720	3.78 (3.51-4.07)	3.7 (3.44-3.98)	1417.38	1.88 (1.63)	3.68 (3.41)
10027433	Metabolism and nutrition disorders	1635	4.06 (3.86-4.27)	3.85 (3.68-4.04)	3483.30	1.94 (1.77)	3.83 (3.64)
10022117	Injury, poisoning and procedural complications	213	13.54 (11.81-15.53)	13.44 (11.73-15.39)	2373.91	3.7 (3.25)	13.03 (11.36)
10014698	Endocrine disorders	289	7.69 (6.84-8.65)	7.61 (6.78-8.55)	1631.98	2.91 (2.51)	7.49 (6.66)
10028395	Musculoskeletal and connective tissue disorders	337	14.85 (13.31-16.57)	14.66 (13.16-16.34)	4143.76	3.83 (3.46)	14.18 (12.71)
10040785	Skin and subcutaneous tissue disorders	446	4.89 (4.45-5.37)	4.81 (4.39-5.28)	1337.11	2.25 (1.94)	4.77 (4.34)
10007541	Cardiac disorders	931	4.18 (3.91-4.47)	4.06 (3.81-4.33)	2146.24	2.01 (1.79)	4.03 (3.77)
10047065	Vascular disorders	409	4.66 (4.23-5.14)	4.6 (4.18-5.07)	1144.16	2.19 (1.86)	4.56 (4.13)
10029205	Nervous system disorders	400	3.56 (3.22-3.93)	3.51 (3.19-3.87)	716.40	1.8 (1.47)	3.49 (3.16)
10021428	Immune system disorders	358	5.9 (5.31-6.56)	5.83 (5.26-6.47)	1415.85	2.53 (2.17)	5.76 (5.19)
10077536	Product issues	12	6.84 (3.87-12.11)	6.84 (3.87-12.1)	58.85	2.75 (1.01)	6.74 (3.81)
10037175	Psychiatric disorders	102	5.97 (4.91-7.26)	5.95 (4.89-7.23)	413.94	2.55 (1.91)	5.88 (4.83)
10010331	Congenital, familial and genetic disorders	5	17.5 (7.15-42.86)	17.5 (7.15-42.84)	74.54	4.07 (1.54)	16.81 (6.87)
10015919	Eye disorders	4	8.26 (3.07-22.22)	8.26 (3.07-22.22)	25.00	3.02 (0.33)	8.11 (3.01)

The top 40 signal strengths of AEs of Everolimus at preferred terms (PTs) level ranked by EBGM are described in Table 3. Stomatitis, pneumonitis and impaired insulin secretion that are included in the label are usually reported in patients with Everolimus. Of note, unexpected significant AEs, including “biliary ischaemia”, “angiofibroma”, “tuberous sclerosis complex”, “urinary fistula”, “renal lymphocele” and so on, were uncovered in the label. However, rash, fatigue, diarrhea, decreased appetite, nausea, hyperlipemia, and anemia, which are listed in the drug label, did not meet the top 40 signal strength. The results may provide a reference for further updating the AE in the specification of Everolimus. Besides, we screened the top 40 high level terms (HLT) of AEs to replace with classification evaluation by using ROR, PRR, BCPNN, and EBGM according to frequency ranking, and the results are shown in Supplementary Table S1. In addition, it is worth noticing that a higher number of PTs were associated with tumorigenesis and development, including “angiomyolipoma”, “metastatic renal cell carcinoma”, and “endometrial adenocarcinoma”.

**TABLE 3:** The top 40 signal strength of AEs of Everolimus at preferred terms (PTs) level ranked by EBGM in FDA Adverse Event Reporting System (FAERS) source.

**AEs**	**SOC**	**Case Reports**	**ROR (95% CI)**	**PRR (95% CI)**	**IC (IC025)**	**EBGM (EBGM05)**
angiofibroma	Musculoskeletal and connective tissue disorders	12	193.29 (97.1-384.76)	193.19 (97.07-384.48)	7.03 (4.97)	130.86 (65.74)
accidental exposure to product packaging	Injury, poisoning and procedural complications	32	112.14 (75.78-165.95)	112 (75.71-165.67)	6.46 (5.21)	87.83 (59.35)
biliary ischaemia	Vascular disorders	6	219.59 (81.2-593.82)	219.54 (81.2-593.58)	7.15 (4.33)	142.41 (52.66)
neuroendocrine tumour	Neoplasms benign, malignant and unspecified (incl cysts and polyps)	109	72.71 (59.28-89.19)	72.39 (59.06-88.74)	5.94 (5.27)	61.51 (50.14)
angiomyolipoma	Musculoskeletal and connective tissue disorders	14	80.54 (45.37-142.99)	80.5 (45.36-142.87)	6.07 (4.29)	67.25 (37.88)
tuberous sclerosis complex	Neoplasms benign, malignant and unspecified (incl cysts and polyps)	9	92.91 (45-191.83)	92.88 (45-191.72)	6.24 (4.06)	75.65 (36.64)
pancreatic neuroendocrine tumour	Endocrine disorders	34	59.84 (41.73-85.81)	59.76 (41.69-85.65)	5.7 (4.55)	52.16 (36.38)
urinary fistula	Renal and urinary disorders	7	90.91 (40.03-206.47)	90.88 (40.02-206.37)	6.22 (3.79)	74.33 (32.73)
concomitant disease progression	General disorders and administration site conditions	78	42.21 (33.42-53.32)	42.08 (33.34-53.12)	5.26 (4.49)	38.19 (30.24)
stomatitis	Gastrointestinal disorders	1695	35.2 (33.44-37.04)	32.84 (31.3-34.45)	4.93 (4.76)	30.44 (28.92)
neuroendocrine tumour of the lung	Endocrine disorders	6	80.52 (33.51-193.46)	80.5 (33.51-193.38)	6.07 (3.52)	67.25 (27.99)
lymphocele	Blood and lymphatic system disorders	22	47.9 (30.78-74.55)	47.86 (30.77-74.45)	5.42 (4.02)	42.88 (27.56)
lymphangioleiomyomatosis	Blood and lymphatic system disorders	8	67.1 (31.74-141.86)	67.08 (31.74-141.78)	5.85 (3.61)	57.64 (27.27)
hepatic artery stenosis	Vascular disorders	6	58.91 (25.01-138.78)	58.9 (25.01-138.72)	5.69 (3.19)	51.51 (21.87)
renal lymphocele	Blood and lymphatic system disorders	3	92.89 (26.47-326)	92.88 (26.47-325.92)	6.24 (2.88)	75.65 (21.56)
metastases to pleura	Neoplasms benign, malignant and unspecified (incl cysts and polyps)	27	34.21 (23.09-50.68)	34.17 (23.08-50.61)	4.98 (3.72)	31.58 (21.31)
pneumonitis	Respiratory, thoracic and mediastinal disorders	531	25.04 (22.92-27.36)	24.52 (22.48-26.74)	4.53 (4.24)	23.17 (21.21)
human herpesvirus 7 infection	Infections and infestations	4	64.41 (22.41-185.08)	64.4 (22.41-185.03)	5.8 (2.86)	55.65 (19.37)
lymphangiosis carcinomatosa	Blood and lymphatic system disorders	27	29.89 (20.21-44.19)	29.85 (20.2-44.13)	4.8 (3.55)	27.86 (18.84)
thoracic cavity drainage	Surgical and medical procedures	9	38.96 (19.65-77.24)	38.95 (19.65-77.2)	5.15 (3.09)	35.6 (17.96)
metastatic renal cell carcinoma	Renal and urinary disorders	52	25.15 (19-33.29)	25.09 (18.97-33.2)	4.57 (3.65)	23.68 (17.89)
rhabdomyoma	Musculoskeletal and connective tissue disorders	3	71.04 (20.82-242.41)	71.03 (20.82-242.35)	5.92 (2.64)	60.52 (17.74)
incisional hernia repair	Surgical and medical procedures	4	57.51 (20.17-163.96)	57.5 (20.17-163.91)	5.66 (2.75)	50.44 (17.69)
liver transplant rejection	Injury, poisoning and procedural complications	43	25.53 (18.75-34.76)	25.49 (18.73-34.68)	4.59 (3.59)	24.03 (17.65)
metastases to kidney	Neoplasms benign, malignant and unspecified (incl cysts and polyps)	18	30.59 (18.94-49.4)	30.57 (18.93-49.35)	4.83 (3.32)	28.48 (17.63)
impaired insulin secretion	Endocrine disorders	5	49.09 (19.4-124.24)	49.08 (19.4-124.2)	5.45 (2.81)	43.86 (17.33)
renal hamartoma	Renal and urinary disorders	5	47.92 (18.96-121.14)	47.91 (18.96-121.1)	5.42 (2.78)	42.92 (16.98)
cholangiolitis	Hepatobiliary disorders	3	67.09 (19.76-227.77)	67.08 (19.76-227.72)	5.85 (2.59)	57.64 (16.98)
oncologic complication	Neoplasms benign, malignant and unspecified (incl cysts and polyps)	30	25.67 (17.74-37.13)	25.64 (17.73-37.07)	4.59 (3.41)	24.16 (16.7)
abdominal hernia repair	Surgical and medical procedures	6	42.38 (18.27-98.29)	42.37 (18.27-98.25)	5.26 (2.82)	38.43 (16.57)
oral pain	Gastrointestinal disorders	403	19.16 (17.32-21.19)	18.86 (17.08-20.82)	4.17 (3.84)	18.06 (16.33)
metastatic carcinoid tumour	Neoplasms benign, malignant and unspecified (incl cysts and polyps)	6	41.65 (17.97-96.53)	41.64 (17.97-96.49)	5.24 (2.8)	37.83 (16.32)
lymphatic fistula	Injury, poisoning and procedural complications	3	63.56 (18.81-214.8)	63.55 (18.81-214.74)	5.78 (2.53)	55.02 (16.28)
endometrial adenocarcinoma	Neoplasms benign, malignant and unspecified (incl cysts and polyps)	16	28.39 (17.1-47.14)	28.37 (17.09-47.09)	4.73 (3.15)	26.57 (16)
extubation	Surgical and medical procedures	4	50.32 (17.79-142.29)	50.31 (17.79-142.25)	5.49 (2.6)	44.83 (15.85)
drain removal	Surgical and medical procedures	3	60.38 (17.94-203.21)	60.37 (17.94-203.16)	5.72 (2.48)	52.63 (15.64)
cell marker increased	Investigations	16	27.54 (16.59-45.7)	27.52 (16.59-45.66)	4.69 (3.11)	25.82 (15.56)
metastases to liver	Hepatobiliary disorders	290	18.38 (16.33-20.69)	18.17 (16.17-20.43)	4.12 (3.73)	17.43 (15.49)
heart transplant rejection	Cardiac disorders	22	25.25 (16.41-38.85)	25.23 (16.4-38.8)	4.57 (3.21)	23.8 (15.47)
pancreatic neuroendocrine tumour metastatic	Endocrine disorders	7	35.67 (16.47-77.28)	35.66 (16.47-77.24)	5.04 (2.76)	32.84 (15.16)

## 4 Discussion

The spontaneous reporting system (SRS) has been widely conducted for exploring post-marketing drug safety and assessing suspected AEs. As a web-based and interactive database, FAERS has enabled the detection, assessment, and prevention of adverse effects or other drug-related problems aiming to monitor the safety of pharmacotherapy and enhance patient compliance. Our study conducted the most comprehensive and systematic post-marketing pharmacovigilance analysis of Everolimus-related AEs based on the FAERS, which is intended to provide a scientific reference for clinical rational administration.

In our study, 24,575 reports of Everolimus as the primary suspected drug were identified, nearly half of which came from the United States (47.56%), and the rest from other countries, including Japan (4.47%) and Germany (4.12%). The proportion of AEs occurred in female patients was higher than that in male patients, suggesting that the adverse reactions of Everolimus may be gender-related or possibly due to the indications of Everolimus for advanced hormone receptor-positive breast cancer. Our results have demonstrated a higher rate of AEs in patients aged 40–80 years (34.98%), which coincides with the increased incidence for the age range of breast cancer and renal cell carcinoma in its indications. However, it is crucial to note that age-related information was missing for more than half of the patients (56.46%), which may lead to the deviation of the results.

The most common and significant AEs at SOC levels were “Gastrointestinal disorders”, which are one of the most common AEs of Everolimus in the label and were confirmed significantly in the present study. Stomatitis is one of the most frequently stated reasons for treatment delay or dose reduction among patients receiving Everolimus treatment. According to the BOLERO-2 study, the incidence of oral mucositis is as high as 58% and 81% among Asian patients, and most adverse reactions were grade 1 or 2 (Noguchi et al., 2014). Studies indicated that efficient professional oral care could reduce the incidence and severity of oral mucositis and improve the condition of patients’ oral cavities (Umeda et al., 2021). Notably, significant AEs signal strengths among SOC of “Neoplasms benign, malignant and unspecified (incl cysts and polyps)”, and “Musculoskeletal and connective tissue disorders” were presented in this study, including “pancreatic neuroendocrine tumour”, “metastases to pleura”, “angiomyolipoma”, and “tuberous sclerosis complex”, *etc.* Since Everolimus has been widely prescribed for a long time for the treatment of cancers and connective tissue diseases, it cannot be directly inferred whether benign, malignant, and unspecified tumors and various musculoskeletal and connective tissue diseases ADE are directly caused by Everolimus. In addition, we also analyzed some SOCs unrelated to medical treatment, such as “Investigations (SOC: 10022891,3834)”, which were not further discussed in this study.

Hematological toxicity was not common among patients treated with Everolimus. However, in our study, the signal of blood and lymphatic system disorders were detected and several new PTs were found with significant signal strength, including lymphocele and lymphangiosis carcinomatosa. As an immunosuppressive agent, mTOR inhibitors exert dose-dependent effects on haematopoiesis, potentially leading to anaemia (Sánchez Fructuoso et al., 2007), and the inhibition of signal transduction *via* glycoprotein 130 (β) chain shared by certain cytokine receptors, granulocyte colony-stimulating factor and erythropoietin may induce leukopenia and thrombocytopenia (Molina et al., 2012). Decreased hemoglobin, lymphocytes, and platelets have been reported in clinical trials (Chocteau-Bouju et al., 2015; Kanesvaran et al., 2015), thus monitoring of complete blood count is recommended prior to the start of Everolimus therapy and periodically thereafter, and our results have re-emphasized the importance of the haematological observations. Drug-related pneumonitis is one of the potential adverse side effects of cancer treatments, which is characterized clinically by difficulty of breathing and dry, non-productive cough and presented radiologically as different radiographic manifestations. The reported incidence of Everolimus-related pneumonitis ranges from 13.5% to 48.7% (Penttilä et al., 2017), and the proposed etiologic factors include autoimmune response and delayed-type hypersensitivity (Garrean et al., 2005). However, studies have shown that Everolimus-induced pneumonitis is associated with improved survival in tumor patients and may serve as a biomarker of Everolimus efficacy, which means that adequate management of this adverse event may be reinforced with the goal of preserving Everolimus therapy when possible (Taboada et al., 2022).

Other AEs of SOCs involved in adverse reactions mentioned in the instructions, including Infections and Endocrine disorders, have corresponding signals detected and verified the reliability of the data in this study. Some other unexpected and new significant AEs signals that were not mentioned in the instruction or regulatory trials, such as urinary fistula and hepatic artery stenosis, were detected in our analysis, and the exact induction mechanisms of these AEs remained unclear. However, it has to be emphasized that Everolimus has a narrow therapeutic window and high pharmacokinetic variability within and between individuals, and the toxicity of drugs is closely related to the dosage and duration of medication. Therefore, during the Everolimus treatment, therapeutic drug monitoring was recommended to avoid under-immunosuppression or over-immunosuppression and to minimize the occurrence of adverse effects, especially in the transplantation setting (Shipkova et al., 2016). In addition, in our investigation we only studied the correlation between the Everolimus and AEs in advance, so the drug role we set is “preferred suspect”, that is, the correlation between the drug as a single factor and AEs. As a result, the influence of multi-factor confounding was not further investigated in our study, including secondary suspect, drug concomitant, and multi-drug interactions.

This real-world observational study with large-sample data has some limitations which need to be acknowledged. First, all of the AEs and medication error reports are voluntarily submitted by the pharmaceutical industry, healthcare providers, and consumers, thus there may be a risk of missing data or information bias. Second, definite causality is not required compulsorily when data was uploaded, so the reports can only provide safety signal strength but do not represent a real risk. Finally, due to the lack of total numbers of patients treated with Everolimus, the true incidence of ae cannot be calculated from FAERS. Prospective, large-scale, and longer-term clinical studies are still needed for further validation.

## 5 Conclusion

Our pharmacovigilance analysis of FAERS database comprehensively and systematically revealed the safety signals in treatment with Everolimus. More attention should be given to the new and unexpected AE events when medication is administered, and several life-threatening AEs warrant early detection and intervention. Long-term drug monitoring and scientifically designed prospective trials are encouraged to confirm these results in future studies.

## Data Availability

Publicly available datasets were analyzed in this study. This data can be found here: https://www.fda.gov/.
