# Supplementary material for: A real-world disproportionality analysis of Everolimus: data mining of the public version of FDA adverse event reporting system
Source: Front Pharmacol. 2024 Mar 12;15:1333662. doi: 10.3389/fphar.2024.1333662 (PMC10964017; doi:10.3389/fphar.2024.1333662)
Supplement: Supplementary file 1 [file Table1.DOCX]

Supplementary Material

# Supplementary Figures and Tables

## Supplementary Table 1

| High Level Term (HLT) | Case Reports | ROR_95 | PRR_95 | IC(IC025) | EBGM(EBGM05) |
| --- | --- | --- | --- | --- | --- |
| Death and sudden death | 4373 | 4.32 (4.18-4.46) | 3.73 (3.63-3.83) | 1.89 (1.78) | 3.7 (3.58) |
| Neoplasms malignant site unspecified NEC | 2292 | 11.64 (11.15-12.16) | 10.65 (10.24-11.08) | 3.38 (3.23) | 10.4 (9.96) |
| Stomatitis and ulceration | 2089 | 28.75 (27.45-30.1) | 26.39 (25.29-27.53) | 4.63 (4.48) | 24.83 (23.71) |
| General signs and symptoms NEC | 1619 | 4.37 (4.15-4.6) | 4.15 (3.96-4.35) | 2.04 (1.87) | 4.12 (3.91) |
| Diarrhoea (excl infective) | 1537 | 2.59 (2.46-2.73) | 2.49 (2.38-2.62) | 1.31 (1.14) | 2.48 (2.36) |
| Appetite disorders | 1018 | 4.1 (3.85-4.37) | 3.97 (3.74-4.22) | 1.98 (1.77) | 3.94 (3.7) |
| Febrile disorders | 883 | 2.94 (2.75-3.15) | 2.87 (2.69-3.07) | 1.52 (1.29) | 2.86 (2.67) |
| Coughing and associated symptoms | 786 | 3.12 (2.91-3.35) | 3.05 (2.85-3.27) | 1.6 (1.36) | 3.04 (2.83) |
| Lower respiratory tract infections NEC | 746 | 2.42 (2.25-2.6) | 2.38 (2.21-2.55) | 1.24 (1) | 2.37 (2.2) |
| Physical examination procedures and organ system status | 646 | 2.69 (2.49-2.91) | 2.65 (2.45-2.86) | 1.4 (1.14) | 2.64 (2.44) |
| Lower respiratory tract inflammatory and immunologic conditions | 563 | 22.7 (20.83-24.73) | 22.2 (20.41-24.14) | 4.4 (4.11) | 21.09 (19.36) |
| Oral soft tissue signs and symptoms | 561 | 12.27 (11.27-13.36) | 12.01 (11.06-13.05) | 3.55 (3.26) | 11.69 (10.74) |
| Heart failure signs and symptoms | 549 | 3.89 (3.57-4.23) | 3.82 (3.52-4.15) | 1.92 (1.64) | 3.8 (3.49) |
| Neoplasms unspecified malignancy and site unspecified NEC | 496 | 8.82 (8.07-9.65) | 8.67 (7.93-9.46) | 3.09 (2.79) | 8.5 (7.77) |
| Red blood cell analyses | 495 | 3.75 (3.43-4.1) | 3.69 (3.38-4.03) | 1.87 (1.57) | 3.67 (3.35) |
| Anaemias NEC | 490 | 2.88 (2.63-3.15) | 2.84 (2.6-3.1) | 1.5 (1.2) | 2.82 (2.58) |
| Renal function analyses | 436 | 5.19 (4.72-5.71) | 5.12 (4.66-5.62) | 2.34 (2.02) | 5.07 (4.61) |
| Pneumothorax and pleural effusions NEC | 429 | 6.16 (5.59-6.78) | 6.07 (5.52-6.67) | 2.58 (2.26) | 5.99 (5.44) |
| Respiratory tract disorders NEC | 369 | 6.3 (5.68-6.99) | 6.22 (5.62-6.89) | 2.62 (2.27) | 6.14 (5.54) |
| Total fluid volume decreased | 364 | 3.25 (2.93-3.61) | 3.22 (2.91-3.57) | 1.68 (1.33) | 3.2 (2.89) |
| Sensory abnormalities NEC | 349 | 3.31 (2.98-3.68) | 3.28 (2.95-3.64) | 1.71 (1.35) | 3.26 (2.93) |
| Haemorrhages NEC | 340 | 4.41 (3.96-4.92) | 4.37 (3.93-4.86) | 2.11 (1.75) | 4.33 (3.89) |
| Vascular tests NEC (incl blood pressure) | 339 | 2.41 (2.16-2.68) | 2.39 (2.15-2.65) | 1.25 (0.89) | 2.38 (2.14) |
| Immune and associated conditions NEC | 334 | 6.14 (5.51-6.85) | 6.07 (5.46-6.76) | 2.58 (2.22) | 6 (5.38) |
| Platelet analyses | 293 | 2.96 (2.63-3.32) | 2.93 (2.62-3.29) | 1.54 (1.16) | 2.92 (2.6) |
| Malignant hepatobiliary neoplasms | 290 | 18.38 (16.33-20.69) | 18.17 (16.17-20.43) | 4.12 (3.73) | 17.43 (15.49) |
| Malignant musculoskeletal and connective tissue neoplasms | 277 | 15.01 (13.31-16.93) | 14.85 (13.18-16.73) | 3.84 (3.44) | 14.36 (12.73) |
| Therapeutic procedures NEC | 249 | 2.99 (2.64-3.39) | 2.97 (2.63-3.37) | 1.57 (1.15) | 2.96 (2.61) |
| Hepatobiliary function diagnostic procedures | 222 | 3.84 (3.36-4.38) | 3.81 (3.34-4.35) | 1.92 (1.48) | 3.79 (3.32) |
| Mucosal findings abnormal | 221 | 10.09 (8.82-11.54) | 10.01 (8.76-11.43) | 3.29 (2.84) | 9.79 (8.56) |
| Acnes | 216 | 6.12 (5.34-7) | 6.07 (5.31-6.94) | 2.58 (2.13) | 6 (5.24) |
| Protein analyses NEC | 216 | 6.38 (5.57-7.3) | 6.33 (5.54-7.24) | 2.64 (2.19) | 6.25 (5.46) |
| Lower respiratory tract neoplasms | 215 | 14.54 (12.69-16.67) | 14.42 (12.6-16.52) | 3.8 (3.35) | 13.96 (12.18) |
| Parenchymal lung disorders NEC | 214 | 7.82 (6.82-8.95) | 7.76 (6.78-8.88) | 2.93 (2.48) | 7.63 (6.66) |
| Oedema NEC | 213 | 3.53 (3.09-4.05) | 3.51 (3.07-4.02) | 1.8 (1.35) | 3.49 (3.05) |
| Renal failure and impairment | 209 | 2.33 (2.03-2.67) | 2.32 (2.03-2.66) | 1.21 (0.76) | 2.31 (2.02) |
| Mineral and electrolyte analyses | 201 | 3.27 (2.85-3.76) | 3.25 (2.83-3.74) | 1.69 (1.23) | 3.24 (2.81) |
| Left ventricular failures | 199 | 3.84 (3.34-4.42) | 3.81 (3.32-4.38) | 1.92 (1.45) | 3.79 (3.29) |
| Renal neoplasms | 198 | 16.65 (14.44-19.2) | 16.52 (14.34-19.04) | 3.99 (3.52) | 15.91 (13.8) |
| Hyperglycaemic conditions NEC | 196 | 5.86 (5.09-6.75) | 5.82 (5.06-6.7) | 2.52 (2.05) | 5.75 (4.99) |

**Supplementary Table 1.** The top 40 high level terms (HLT) of AEs evaluated using ROR, PRR, BCPNN, and EBGM according to frequency ranking.
